# {2-[1-(2-Meth­oxy-6-oxidophenyl-κ*O* ^6^)ethyl­idene]-*N*-methyl­hydrazinecarbo­thio­amidato-κ^2^ *N* ^2^,*S*}(tri­phenyl­phosphane-κ*P*)palladium(II) ethanol monosolvate

**DOI:** 10.1107/S1600536813023040

**Published:** 2013-09-04

**Authors:** Brian J. Anderson, Kelly A. O’Rourke, Alexander M. Keeler, Jerry P. Jasinski

**Affiliations:** a Department of Chemistry, Keene State College, 229 Main Street, Keene, NH 03435-2001, USA

## Abstract

In the title compound, [Pd(C_11_H_13_N_3_O_2_S)(C_18_H_15_P)]·C_2_H_5_OH, the Pd^II^ atom is tetra­coordinated in a slightly distorted square-planar environment by three donor atoms (NOS) from a thio­semicarbazonate ligand, forming five- and six-membered chelate rings, and a P atom from a neutral tri­phenyl­phosphane group. The five-membered ring adopts a distorted envelope conformation with Pd^II^ as the flap atom, while the six-membered ring forms a slightly twisted screw-boat conformation. A slightly distorted screw-boat form of a meth­oxy­phenyl group is fused to the six-membered ring. Weak C—H⋯O inter­actions form dimers in the asymmetric unit and along [001] which help to stabilize the crystal packing.

## Related literature

For multiple binding modes of thio­semicarbazones, see: Lobana *et al.* (2009 ▶). For the synthesis of thio­semicarbazone complexes, see: Lobana *et al.* (2012 ▶). For palladium thio­semicarbazone complexes, see: Chellan *et al.* (2010 ▶). For comparison with the anti-cancer drug cisplatin, see: Halder *et al.* (2008 ▶). For puckering parameters, see: Cremer & Pople (1975 ▶). For standard bond lengths, see: Allen *et al.* (1987 ▶).
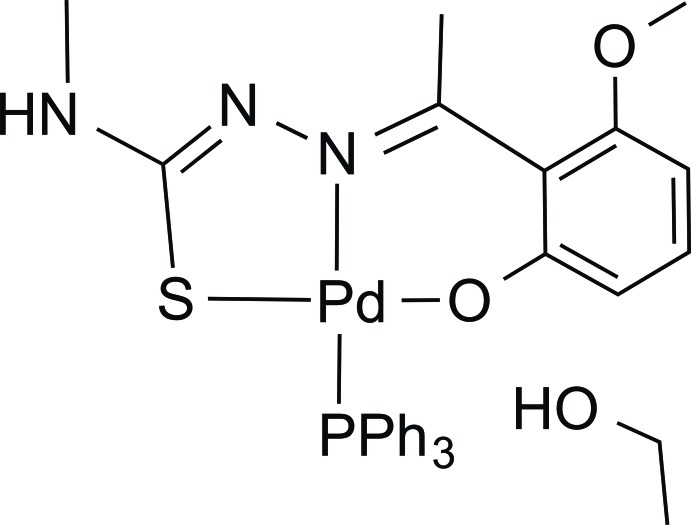


## Experimental

### 

#### Crystal data

[Pd(C_11_H_13_N_3_O_2_S)(C_18_H_15_P)]·C_2_H_6_O
*M* *_r_* = 666.04
Triclinic, 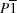

*a* = 8.0294 (6) Å
*b* = 12.0187 (6) Å
*c* = 16.2151 (8) Å
α = 105.764 (4)°
β = 100.835 (5)°
γ = 94.965 (5)°
*V* = 1463.33 (15) Å^3^
*Z* = 2
Mo *K*α radiation
μ = 0.80 mm^−1^
*T* = 173 K
0.28 × 0.12 × 0.06 mm

#### Data collection

Agilent Xcalibur (Eos, Gemini) diffractometer
Absorption correction: multi-scan (*CrysAlis PRO* and *CrysAlis RED*; Agilent, 2012 ▶) *T*_min_ = 0.744, *T*_max_ = 1.000
17885 measured reflections
9710 independent reflections
8289 reflections with *I* > 2σ(*I*)
*R*_int_ = 0.049

#### Refinement

*R*[*F* ^2^ > 2σ(*F* ^2^)] = 0.049
*wR*(*F* ^2^) = 0.128
*S* = 1.10
9710 reflections
366 parameters
H-atom parameters constrained
Δρ_max_ = 2.09 e Å^−3^
Δρ_min_ = −1.04 e Å^−3^

### 

Data collection: *CrysAlis PRO* (Agilent, 2012 ▶); cell refinement: *CrysAlis PRO*; data reduction: *CrysAlis RED* (Agilent, 2012 ▶); program(s) used to solve structure: *SUPERFLIP* (Palatinus & Chapuis, 2007 ▶); program(s) used to refine structure: *SHELXL2012* (Sheldrick, 2008 ▶); molecular graphics: *XP* in *SHELXTL* (Sheldrick, 2008 ▶); software used to prepare material for publication: *OLEX2* (Dolomanov *et al.*, 2009 ▶).

## Supplementary Material

Crystal structure: contains datablock(s) I. DOI: 10.1107/S1600536813023040/bv2224sup1.cif

Structure factors: contains datablock(s) I. DOI: 10.1107/S1600536813023040/bv2224Isup2.hkl

Additional supplementary materials:  crystallographic information; 3D view; checkCIF report

## Figures and Tables

**Table 1:** Hydrogen-bond geometry (Å, °)

*D*—H⋯*A*	*D*—H	H⋯*A*	*D*⋯*A*	*D*—H⋯*A*
C9—H9*C*⋯O2^i^	0.98	2.69	3.439 (5)	133

Symmetry code: (i) 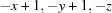.

**Fig. 1.**
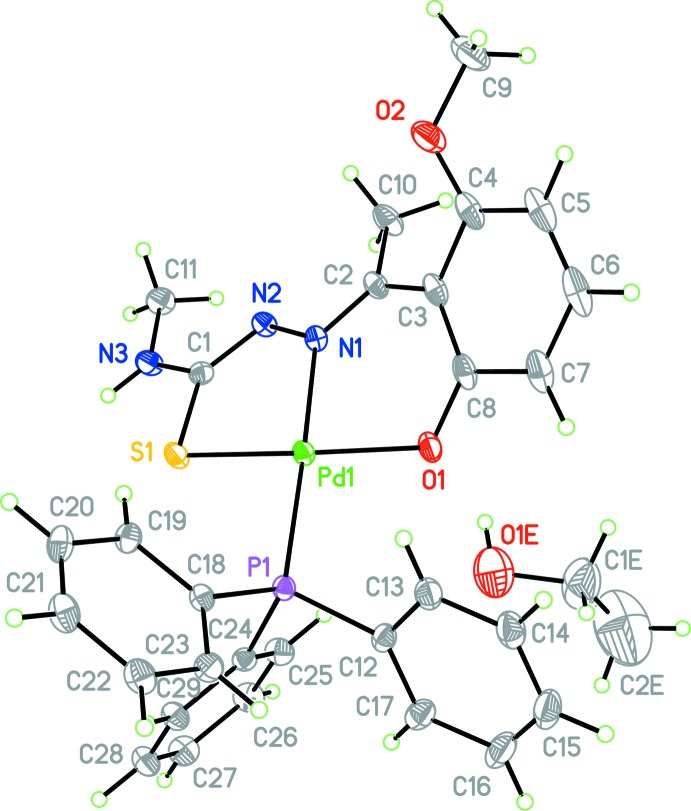
Molecular structure of the title compound showing the atom labeling scheme and 30% probability displacement ellipsoids.

**Fig. 2.**
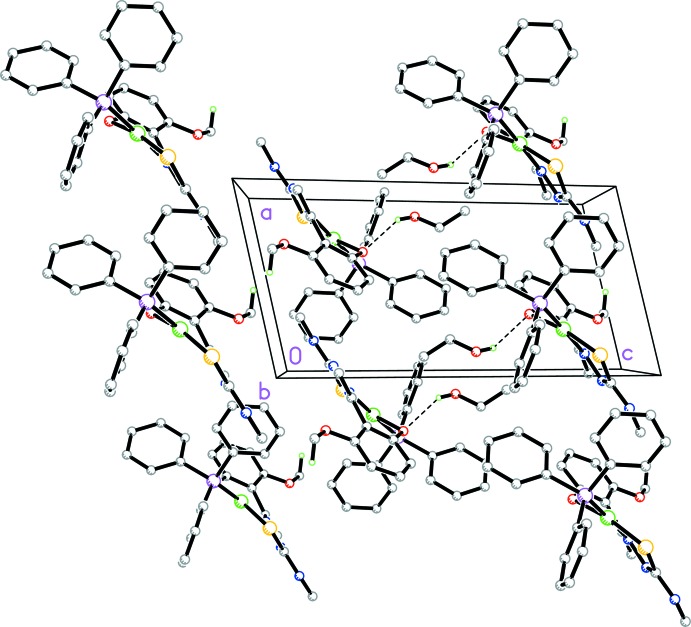
Packing diagram of the title compound viewed along the b axis. Dashed lines indicate weak C9—H9c···O2 intermolecular interactions between the hydroxy-O oxygen atom and the ethanol solvate group forming dimers in the asymmetric unit and along [001]. H atoms not involved in these intermolecular interactions have been omitted for clarity.

**Fig. 3.**
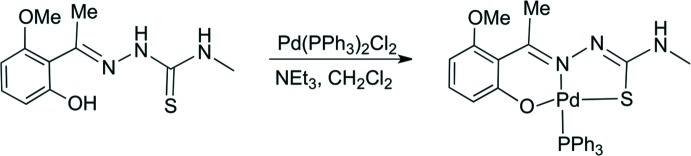
Synthetic scheme of (I).

## supplementary crystallographic information

### 1. Comment

Thiosemicarbazones are a versatile class of ligands that can adopt multiple modes of binding to a metal (Lobana *et al.*, 2009). The synthesis and structure determination of these metal complexes is an active area of research (Lobana *et al.*, 2012). Palladium complexes with thiosemicarbazone ligands, in particular, have been shown to have a variety of biological properties including anti-fungal and anti-tumor activity (Chellan *et al.*, 2010). A recent study compared the cytotoxic effects of a palladium thiosemicarbazone complex to be comparable to the anti-cancer drug cisplatin (Halder *et al.*, 2008). In view of the importance of these types of complexes, we report here in the synthesis and crystal structure of the title compound, C_29_H_28_N_3_O_2_PPdS, C_2_H_6_O, (I).

In (I) the palladium atom is in a slightly distorted square planar environment tetra-coordinated by three donor atoms (NOS) from a thiosemicarbazonate ligand, L1 (C_11_H_13_N_3_O_2_S), forming 5 (Pd1/S1/C1/N2/N1) and 6-membered (Pd1/O1/C8/C3/C2/N1) chelate rings and a phosphorous atom from a neutral triphenyl phosphane group (Fig. 1). The 5-membered ring adopts a distorted envelope conformation while the 6-membered ring forms a slightly twisted screw-boat conformation with puckering parameters Q = 0.2509 (5)Å, φ = 177.7 (5)° and Q = 0.506 (2)Å, θ = 102.5 (2)°, φ = 206.8 (3)°, respectively (Cremer & Pople, 1975). A slightly distorted screw-boat form of a 6-methoxyphenyl group (Q = 0.089 (3)Å, θ = 108 (3)°, φ = 152 (3)°) is fused to the 6-membered ring. Bond lengths are in normal ranges (Allen *et al.*, 1987). Additional weak C—H···O intermolecular interactions are observed forming dimers in the asymmetric unit and along [001] which help stabilize crystal packing (Fig. 2).

### 2. Experimental

The thiosemicarbazone ligand (0.050 g, 0.20 mmol, 1 equiv) was charged to a 50 mL round bottom flask and dissolved in 8 mL of ethanol. The solution was heated under N_2_ to 333° K and triethylamine (0.059 mL, 0.42 mmol, 2.1 equiv) was added via syringe. Pd(PPh_3_)_2_Cl_2_ (0.140 g, 0.20 mmol, 1 equiv) was added to the resulting solution as a solid and the mixture was stirred for seven days. Hexanes, 5 mL, were added and the solution was cooled to 273° K resulting in the formation of a golden yellow solid (Fig. 3). The solid was collected by vacuum filtration and then dissolved in minimal dichloromethane layered with hexanes and stored at 273° K for 1 week resulting in the formation of bright orange single crystals of the title compound. (m.p.: 421–423 K).

### 3. Refinement

All of the H atoms were placed in their calculated positions and then refined using the riding model with atom—H lengths of 0.95Å (CH), 0.99Å (CH_2_) or 0.88° (NH). Isotropic displacement parameters for these atoms were set to 1.2 (CH, NH) or 1.5 (CH_3_, OH) times *U*_eq_ of the parent atom. Idealised Me refined as rotating group: C9(H9A,H9B,H9C), C10(H10A,H10B,H10C), C11(H11A,H11B,H11C), C2E(H2EA,H2EB,H2EC. Idealised tetrahedral OH refined as rotating group O1E(H1E).

### Crystal data

**Table:**

[Pd(C_11_H_13_N_3_O_2_S)(C_18_H_15_P)]·C_2_H_6_O	*Z* = 2
*M**_r_* = 666.04	*F*(000) = 684
Triclinic, *P*1	*D*_x_ = 1.512 Mg m^−^^3^
*a* = 8.0294 (6) Å	Mo *K*α radiation, λ = 0.71073 Å
*b* = 12.0187 (6) Å	Cell parameters from 6938 reflections
*c* = 16.2151 (8) Å	θ = 2.9–32.8°
α = 105.764 (4)°	µ = 0.80 mm^−^^1^
β = 100.835 (5)°	*T* = 173 K
γ = 94.965 (5)°	Irregular, orange
*V* = 1463.33 (15) Å^3^	0.28 × 0.12 × 0.06 mm

### Data collection

**Table:**

Agilent Xcalibur (Eos, Gemini) diffractometer	9710 independent reflections
Radiation source: Enhance (Mo) X-ray Source	8289 reflections with *I* > 2σ(*I*)
Graphite monochromator	*R*_int_ = 0.049
Detector resolution: 16.0416 pixels mm^-1^	θ_max_ = 32.9°, θ_min_ = 2.9°
ω scans	*h* = −12→11
Absorption correction: multi-scan (*CrysAlis PRO* and *CrysAlis RED*; Agilent, 2012)	*k* = −17→17
*T*_min_ = 0.744, *T*_max_ = 1.000	*l* = −23→17
17885 measured reflections	

### Refinement

**Table:**

Refinement on *F*^2^	0 restraints
Least-squares matrix: full	Hydrogen site location: inferred from neighbouring sites
*R*[*F*^2^ > 2σ(*F*^2^)] = 0.049	H-atom parameters constrained
*wR*(*F*^2^) = 0.128	*w* = 1/[σ^2^(*F*_o_^2^) + (0.059*P*)^2^ + 0.4504*P*] where *P* = (*F*_o_^2^ + 2*F*_c_^2^)/3
*S* = 1.10	(Δ/σ)_max_ < 0.001
9710 reflections	Δρ_max_ = 2.09 e Å^−^^3^
366 parameters	Δρ_min_ = −1.04 e Å^−^^3^

### Special details

**Table:**

Geometry. All esds (except the esd in the dihedral angle between two l.s. planes) are estimated using the full covariance matrix. The cell esds are taken into account individually in the estimation of esds in distances, angles and torsion angles; correlations between esds in cell parameters are only used when they are defined by crystal symmetry. An approximate (isotropic) treatment of cell esds is used for estimating esds involving l.s. planes.

### Fractional atomic coordinates and isotropic or equivalent isotropic displacement parameters (Å^2^)

**Table:**

	*x*	*y*	*z*	*U*_iso_*/*U*_eq_	
Pd1	0.75727 (2)	0.18506 (2)	0.21974 (2)	0.02286 (7)	
S1	0.89331 (9)	0.04545 (6)	0.14838 (5)	0.03020 (14)	
P1	0.62186 (8)	0.05981 (5)	0.27706 (4)	0.02202 (12)	
O1	0.6749 (3)	0.32610 (17)	0.29322 (14)	0.0381 (5)	
O2	0.7015 (4)	0.5350 (2)	0.08356 (17)	0.0513 (6)	
N1	0.8907 (2)	0.29209 (18)	0.16787 (13)	0.0220 (4)	
N2	1.0369 (3)	0.25554 (19)	0.13901 (14)	0.0255 (4)	
N3	1.1762 (3)	0.0988 (2)	0.09728 (16)	0.0332 (5)	
H3	1.1826	0.0242	0.0904	0.040*	
C1	1.0436 (3)	0.1445 (2)	0.12762 (16)	0.0254 (5)	
C2	0.8668 (3)	0.3973 (2)	0.17075 (17)	0.0275 (5)	
C3	0.7120 (3)	0.4425 (2)	0.19473 (19)	0.0315 (5)	
C4	0.6409 (4)	0.5239 (2)	0.1547 (2)	0.0402 (7)	
C5	0.5113 (4)	0.5831 (3)	0.1844 (3)	0.0481 (8)	
H5	0.4635	0.6369	0.1568	0.058*	
C6	0.4537 (4)	0.5621 (3)	0.2547 (3)	0.0518 (9)	
H6	0.3719	0.6064	0.2780	0.062*	
C7	0.5120 (4)	0.4784 (3)	0.2918 (3)	0.0465 (8)	
H7	0.4686	0.4654	0.3395	0.056*	
C8	0.6358 (4)	0.4113 (2)	0.2598 (2)	0.0346 (6)	
C9	0.6682 (6)	0.6331 (3)	0.0522 (3)	0.0619 (11)	
H9A	0.7236	0.6310	0.0030	0.093*	
H9B	0.7139	0.7055	0.0997	0.093*	
H9C	0.5443	0.6297	0.0325	0.093*	
C10	1.0040 (4)	0.4756 (3)	0.1521 (2)	0.0397 (7)	
H10A	1.1173	0.4589	0.1763	0.060*	
H10B	0.9948	0.5575	0.1796	0.060*	
H10C	0.9888	0.4610	0.0885	0.060*	
C11	1.3087 (4)	0.1691 (3)	0.0756 (2)	0.0371 (6)	
H11A	1.2649	0.1813	0.0186	0.056*	
H11B	1.4091	0.1285	0.0727	0.056*	
H11C	1.3413	0.2448	0.1209	0.056*	
C12	0.5163 (3)	0.1308 (2)	0.36265 (16)	0.0276 (5)	
C13	0.3869 (4)	0.1960 (2)	0.3403 (2)	0.0351 (6)	
H13	0.3567	0.2009	0.2820	0.042*	
C14	0.3031 (5)	0.2535 (3)	0.4037 (2)	0.0450 (8)	
H14	0.2141	0.2961	0.3882	0.054*	
C15	0.3498 (5)	0.2484 (3)	0.4899 (3)	0.0539 (10)	
H15	0.2944	0.2891	0.5334	0.065*	
C16	0.4764 (5)	0.1842 (3)	0.5119 (2)	0.0509 (9)	
H16	0.5068	0.1802	0.5705	0.061*	
C17	0.5607 (4)	0.1249 (3)	0.44846 (18)	0.0367 (6)	
H17	0.6476	0.0808	0.4640	0.044*	
C18	0.4511 (3)	−0.0493 (2)	0.19927 (15)	0.0239 (4)	
C19	0.4597 (3)	−0.0906 (2)	0.11095 (16)	0.0285 (5)	
H19	0.5546	−0.0619	0.0915	0.034*	
C20	0.3297 (4)	−0.1735 (3)	0.05170 (19)	0.0357 (6)	
H20	0.3360	−0.2009	−0.0082	0.043*	
C21	0.1915 (4)	−0.2166 (3)	0.0789 (2)	0.0384 (6)	
H21	0.1027	−0.2729	0.0379	0.046*	
C22	0.1829 (4)	−0.1773 (3)	0.1667 (2)	0.0358 (6)	
H22	0.0885	−0.2074	0.1858	0.043*	
C23	0.3120 (3)	−0.0940 (2)	0.22670 (18)	0.0301 (5)	
H23	0.3054	−0.0674	0.2866	0.036*	
C24	0.7717 (3)	−0.0240 (2)	0.32316 (15)	0.0239 (4)	
C25	0.9345 (3)	0.0329 (3)	0.37134 (18)	0.0323 (6)	
H25	0.9620	0.1149	0.3828	0.039*	
C26	1.0558 (4)	−0.0296 (3)	0.40240 (19)	0.0372 (6)	
H26	1.1656	0.0100	0.4355	0.045*	
C27	1.0187 (4)	−0.1491 (3)	0.3857 (2)	0.0376 (6)	
H27	1.1025	−0.1916	0.4070	0.045*	
C28	0.8580 (4)	−0.2067 (3)	0.33740 (19)	0.0358 (6)	
H28	0.8322	−0.2889	0.3256	0.043*	
C29	0.7352 (3)	−0.1451 (2)	0.30641 (18)	0.0302 (5)	
H29	0.6255	−0.1853	0.2736	0.036*	
O1E	0.8782 (5)	0.3401 (3)	0.4640 (3)	0.0893 (11)	
H1E	0.9161	0.3693	0.4284	0.134*	
C1E	0.8136 (9)	0.4283 (5)	0.5244 (4)	0.0940 (19)	
H1EA	0.8098	0.4982	0.5034	0.113*	
H1EB	0.6947	0.3986	0.5244	0.113*	
C2E	0.9065 (13)	0.4600 (9)	0.6067 (7)	0.189 (5)	
H2EA	1.0265	0.4837	0.6069	0.283*	
H2EB	0.8986	0.3939	0.6309	0.283*	
H2EC	0.8627	0.5256	0.6426	0.283*	

### Atomic displacement parameters (Å^2^)

**Table:**

	*U*^11^	*U*^22^	*U*^33^	*U*^12^	*U*^13^	*U*^23^
Pd1	0.02281 (10)	0.01824 (9)	0.02616 (10)	0.00125 (6)	0.00610 (7)	0.00437 (7)
S1	0.0342 (3)	0.0207 (3)	0.0407 (4)	0.0057 (2)	0.0186 (3)	0.0098 (2)
P1	0.0209 (3)	0.0204 (3)	0.0224 (3)	0.0003 (2)	0.0042 (2)	0.0036 (2)
O1	0.0535 (13)	0.0203 (9)	0.0438 (12)	0.0069 (9)	0.0243 (10)	0.0055 (8)
O2	0.0657 (17)	0.0353 (12)	0.0558 (15)	0.0157 (12)	0.0058 (12)	0.0205 (11)
N1	0.0165 (8)	0.0214 (9)	0.0264 (10)	0.0027 (7)	0.0045 (7)	0.0045 (7)
N2	0.0213 (9)	0.0243 (10)	0.0323 (11)	0.0034 (8)	0.0072 (8)	0.0099 (8)
N3	0.0348 (12)	0.0313 (12)	0.0440 (13)	0.0121 (10)	0.0216 (10)	0.0173 (10)
C1	0.0248 (11)	0.0278 (12)	0.0255 (11)	0.0039 (9)	0.0073 (9)	0.0096 (9)
C2	0.0256 (11)	0.0194 (11)	0.0331 (13)	−0.0011 (9)	0.0011 (9)	0.0055 (9)
C3	0.0263 (12)	0.0180 (11)	0.0428 (15)	0.0000 (9)	0.0013 (10)	0.0023 (10)
C4	0.0326 (14)	0.0199 (12)	0.0570 (19)	0.0019 (10)	−0.0048 (12)	0.0037 (12)
C5	0.0336 (15)	0.0231 (13)	0.073 (2)	0.0063 (12)	−0.0069 (15)	0.0022 (14)
C6	0.0284 (14)	0.0248 (14)	0.087 (3)	0.0051 (11)	0.0066 (15)	−0.0044 (15)
C7	0.0383 (16)	0.0264 (14)	0.069 (2)	0.0024 (12)	0.0204 (15)	−0.0008 (14)
C8	0.0283 (12)	0.0180 (11)	0.0504 (16)	−0.0021 (10)	0.0106 (11)	−0.0013 (11)
C9	0.077 (3)	0.0365 (18)	0.070 (3)	0.0125 (19)	−0.004 (2)	0.0236 (17)
C10	0.0350 (14)	0.0239 (13)	0.062 (2)	−0.0011 (11)	0.0141 (13)	0.0150 (13)
C11	0.0327 (14)	0.0412 (16)	0.0455 (16)	0.0091 (12)	0.0200 (12)	0.0172 (13)
C12	0.0266 (11)	0.0260 (12)	0.0262 (12)	−0.0031 (9)	0.0084 (9)	0.0014 (9)
C13	0.0387 (15)	0.0275 (13)	0.0383 (15)	0.0027 (11)	0.0137 (11)	0.0053 (11)
C14	0.0454 (18)	0.0301 (14)	0.062 (2)	0.0042 (13)	0.0297 (16)	0.0061 (14)
C15	0.064 (2)	0.0386 (17)	0.056 (2)	−0.0038 (17)	0.0382 (18)	−0.0046 (15)
C16	0.062 (2)	0.052 (2)	0.0300 (15)	−0.0084 (18)	0.0189 (14)	−0.0035 (14)
C17	0.0360 (14)	0.0421 (16)	0.0273 (13)	−0.0033 (12)	0.0072 (10)	0.0051 (11)
C18	0.0210 (10)	0.0220 (11)	0.0255 (11)	0.0018 (8)	0.0012 (8)	0.0050 (8)
C19	0.0249 (11)	0.0302 (13)	0.0270 (12)	0.0035 (10)	0.0040 (9)	0.0042 (9)
C20	0.0333 (13)	0.0341 (14)	0.0298 (13)	0.0038 (11)	0.0006 (10)	−0.0021 (11)
C21	0.0283 (13)	0.0319 (14)	0.0424 (16)	−0.0040 (11)	−0.0046 (11)	0.0013 (12)
C22	0.0261 (12)	0.0308 (13)	0.0447 (16)	−0.0053 (11)	0.0045 (11)	0.0067 (11)
C23	0.0266 (12)	0.0304 (13)	0.0304 (12)	−0.0026 (10)	0.0057 (9)	0.0068 (10)
C24	0.0229 (10)	0.0251 (11)	0.0235 (11)	0.0011 (9)	0.0055 (8)	0.0076 (8)
C25	0.0259 (12)	0.0330 (13)	0.0334 (13)	−0.0043 (10)	−0.0005 (10)	0.0099 (11)
C26	0.0265 (12)	0.0426 (16)	0.0386 (15)	−0.0007 (12)	−0.0028 (10)	0.0136 (12)
C27	0.0313 (13)	0.0441 (17)	0.0384 (15)	0.0109 (12)	0.0030 (11)	0.0150 (12)
C28	0.0374 (14)	0.0273 (13)	0.0403 (15)	0.0047 (11)	0.0028 (11)	0.0100 (11)
C29	0.0257 (12)	0.0267 (12)	0.0347 (13)	−0.0009 (10)	0.0015 (9)	0.0080 (10)
O1E	0.087 (3)	0.071 (2)	0.090 (3)	0.001 (2)	0.008 (2)	0.0023 (19)
C1E	0.101 (4)	0.072 (3)	0.084 (4)	−0.022 (3)	0.029 (3)	−0.012 (3)
C2E	0.180 (11)	0.149 (9)	0.188 (10)	0.051 (8)	−0.037 (8)	0.016 (8)

### Geometric parameters (Å, º)

**Table:**

Pd1—S1	2.2550 (7)	C13—H13	0.9500
Pd1—P1	2.2735 (6)	C13—C14	1.393 (4)
Pd1—O1	2.0309 (19)	C14—H14	0.9500
Pd1—N1	2.049 (2)	C14—C15	1.398 (6)
S1—C1	1.763 (3)	C15—H15	0.9500
P1—C12	1.816 (3)	C15—C16	1.380 (6)
P1—C18	1.825 (2)	C16—H16	0.9500
P1—C24	1.817 (3)	C16—C17	1.404 (4)
O1—C8	1.316 (4)	C17—H17	0.9500
O2—C4	1.366 (4)	C18—C19	1.400 (3)
O2—C9	1.430 (4)	C18—C23	1.395 (3)
N1—N2	1.405 (3)	C19—H19	0.9500
N1—C2	1.285 (3)	C19—C20	1.388 (4)
N2—C1	1.304 (3)	C20—H20	0.9500
N3—H3	0.8800	C20—C21	1.380 (4)
N3—C1	1.352 (3)	C21—H21	0.9500
N3—C11	1.454 (4)	C21—C22	1.389 (4)
C2—C3	1.476 (4)	C22—H22	0.9500
C2—C10	1.513 (4)	C22—C23	1.392 (4)
C3—C4	1.417 (4)	C23—H23	0.9500
C3—C8	1.430 (4)	C24—C25	1.399 (3)
C4—C5	1.397 (5)	C24—C29	1.400 (4)
C5—H5	0.9500	C25—H25	0.9500
C5—C6	1.383 (6)	C25—C26	1.384 (4)
C6—H6	0.9500	C26—H26	0.9500
C6—C7	1.376 (5)	C26—C27	1.382 (4)
C7—H7	0.9500	C27—H27	0.9500
C7—C8	1.420 (4)	C27—C28	1.388 (4)
C9—H9A	0.9800	C28—H28	0.9500
C9—H9B	0.9800	C28—C29	1.384 (4)
C9—H9C	0.9800	C29—H29	0.9500
C10—H10A	0.9800	O1E—H1E	0.8400
C10—H10B	0.9800	O1E—C1E	1.441 (7)
C10—H10C	0.9800	C1E—H1EA	0.9900
C11—H11A	0.9800	C1E—H1EB	0.9900
C11—H11B	0.9800	C1E—C2E	1.334 (10)
C11—H11C	0.9800	C2E—H2EA	0.9800
C12—C13	1.406 (4)	C2E—H2EB	0.9800
C12—C17	1.393 (4)	C2E—H2EC	0.9800
			
S1—Pd1—P1	92.57 (2)	C17—C12—C13	119.6 (3)
O1—Pd1—S1	170.35 (7)	C12—C13—H13	120.0
O1—Pd1—P1	92.90 (6)	C14—C13—C12	120.0 (3)
O1—Pd1—N1	89.83 (8)	C14—C13—H13	120.0
N1—Pd1—S1	84.46 (6)	C13—C14—H14	120.0
N1—Pd1—P1	176.64 (6)	C13—C14—C15	120.1 (3)
C1—S1—Pd1	94.69 (9)	C15—C14—H14	120.0
C12—P1—Pd1	114.01 (9)	C14—C15—H15	120.0
C12—P1—C18	103.25 (11)	C16—C15—C14	120.0 (3)
C12—P1—C24	107.52 (12)	C16—C15—H15	120.0
C18—P1—Pd1	115.52 (8)	C15—C16—H16	119.8
C24—P1—Pd1	110.81 (8)	C15—C16—C17	120.5 (3)
C24—P1—C18	104.95 (11)	C17—C16—H16	119.8
C8—O1—Pd1	119.66 (18)	C12—C17—C16	119.8 (3)
C4—O2—C9	118.9 (3)	C12—C17—H17	120.1
N2—N1—Pd1	117.73 (15)	C16—C17—H17	120.1
C2—N1—Pd1	125.24 (18)	C19—C18—P1	120.04 (18)
C2—N1—N2	116.3 (2)	C23—C18—P1	120.92 (19)
C1—N2—N1	113.4 (2)	C23—C18—C19	119.0 (2)
C1—N3—H3	119.0	C18—C19—H19	120.0
C1—N3—C11	122.1 (2)	C20—C19—C18	120.1 (2)
C11—N3—H3	119.0	C20—C19—H19	120.0
N2—C1—S1	125.8 (2)	C19—C20—H20	119.7
N2—C1—N3	118.5 (2)	C21—C20—C19	120.7 (3)
N3—C1—S1	115.67 (19)	C21—C20—H20	119.7
N1—C2—C3	120.9 (2)	C20—C21—H21	120.2
N1—C2—C10	118.6 (2)	C20—C21—C22	119.7 (3)
C3—C2—C10	120.5 (2)	C22—C21—H21	120.2
C4—C3—C2	118.7 (3)	C21—C22—H22	119.9
C4—C3—C8	118.9 (3)	C21—C22—C23	120.3 (3)
C8—C3—C2	122.4 (3)	C23—C22—H22	119.9
O2—C4—C3	115.5 (3)	C18—C23—H23	119.9
O2—C4—C5	123.3 (3)	C22—C23—C18	120.3 (3)
C5—C4—C3	121.1 (3)	C22—C23—H23	119.9
C4—C5—H5	120.5	C25—C24—P1	118.8 (2)
C6—C5—C4	118.9 (3)	C25—C24—C29	118.6 (2)
C6—C5—H5	120.5	C29—C24—P1	122.35 (19)
C5—C6—H6	119.2	C24—C25—H25	119.8
C7—C6—C5	121.6 (3)	C26—C25—C24	120.4 (3)
C7—C6—H6	119.2	C26—C25—H25	119.8
C6—C7—H7	119.5	C25—C26—H26	119.7
C6—C7—C8	121.0 (3)	C27—C26—C25	120.6 (3)
C8—C7—H7	119.5	C27—C26—H26	119.7
O1—C8—C3	124.6 (3)	C26—C27—H27	120.2
O1—C8—C7	117.8 (3)	C26—C27—C28	119.6 (3)
C7—C8—C3	117.6 (3)	C28—C27—H27	120.2
O2—C9—H9A	109.5	C27—C28—H28	119.8
O2—C9—H9B	109.5	C29—C28—C27	120.4 (3)
O2—C9—H9C	109.5	C29—C28—H28	119.8
H9A—C9—H9B	109.5	C24—C29—H29	119.8
H9A—C9—H9C	109.5	C28—C29—C24	120.4 (2)
H9B—C9—H9C	109.5	C28—C29—H29	119.8
C2—C10—H10A	109.5	C1E—O1E—H1E	109.5
C2—C10—H10B	109.5	O1E—C1E—H1EA	108.8
C2—C10—H10C	109.5	O1E—C1E—H1EB	108.8
H10A—C10—H10B	109.5	H1EA—C1E—H1EB	107.7
H10A—C10—H10C	109.5	C2E—C1E—O1E	113.9 (8)
H10B—C10—H10C	109.5	C2E—C1E—H1EA	108.8
N3—C11—H11A	109.5	C2E—C1E—H1EB	108.8
N3—C11—H11B	109.5	C1E—C2E—H2EA	109.5
N3—C11—H11C	109.5	C1E—C2E—H2EB	109.5
H11A—C11—H11B	109.5	C1E—C2E—H2EC	109.5
H11A—C11—H11C	109.5	H2EA—C2E—H2EB	109.5
H11B—C11—H11C	109.5	H2EA—C2E—H2EC	109.5
C13—C12—P1	117.6 (2)	H2EB—C2E—H2EC	109.5
C17—C12—P1	122.8 (2)		
			
Pd1—S1—C1—N2	−11.1 (2)	C8—C3—C4—O2	−169.7 (2)
Pd1—S1—C1—N3	168.87 (19)	C8—C3—C4—C5	7.2 (4)
Pd1—P1—C12—C13	60.1 (2)	C9—O2—C4—C3	−165.4 (3)
Pd1—P1—C12—C17	−118.6 (2)	C9—O2—C4—C5	17.8 (5)
Pd1—P1—C18—C19	30.2 (2)	C10—C2—C3—C4	36.0 (4)
Pd1—P1—C18—C23	−150.69 (19)	C10—C2—C3—C8	−141.1 (3)
Pd1—P1—C24—C25	41.3 (2)	C11—N3—C1—S1	178.8 (2)
Pd1—P1—C24—C29	−133.5 (2)	C11—N3—C1—N2	−1.2 (4)
Pd1—O1—C8—C3	−29.2 (4)	C12—P1—C18—C19	155.4 (2)
Pd1—O1—C8—C7	152.1 (2)	C12—P1—C18—C23	−25.5 (2)
Pd1—N1—N2—C1	17.1 (3)	C12—P1—C24—C25	−83.9 (2)
Pd1—N1—C2—C3	−14.2 (3)	C12—P1—C24—C29	101.3 (2)
Pd1—N1—C2—C10	163.7 (2)	C12—C13—C14—C15	1.3 (5)
P1—C12—C13—C14	−179.2 (2)	C13—C12—C17—C16	−0.2 (4)
P1—C12—C17—C16	178.5 (2)	C13—C14—C15—C16	−1.4 (5)
P1—C18—C19—C20	−179.9 (2)	C14—C15—C16—C17	0.8 (5)
P1—C18—C23—C22	−179.9 (2)	C15—C16—C17—C12	0.1 (5)
P1—C24—C25—C26	−175.7 (2)	C17—C12—C13—C14	−0.4 (4)
P1—C24—C29—C28	175.1 (2)	C18—P1—C12—C13	−66.0 (2)
O2—C4—C5—C6	177.2 (3)	C18—P1—C12—C17	115.3 (2)
N1—N2—C1—S1	−2.0 (3)	C18—P1—C24—C25	166.7 (2)
N1—N2—C1—N3	178.0 (2)	C18—P1—C24—C29	−8.1 (2)
N1—C2—C3—C4	−146.2 (3)	C18—C19—C20—C21	−0.4 (5)
N1—C2—C3—C8	36.8 (4)	C19—C18—C23—C22	−0.8 (4)
N2—N1—C2—C3	176.2 (2)	C19—C20—C21—C22	−0.5 (5)
N2—N1—C2—C10	−6.0 (3)	C20—C21—C22—C23	0.7 (5)
C2—N1—N2—C1	−172.4 (2)	C21—C22—C23—C18	0.0 (5)
C2—C3—C4—O2	13.1 (4)	C23—C18—C19—C20	1.0 (4)
C2—C3—C4—C5	−170.0 (3)	C24—P1—C12—C13	−176.6 (2)
C2—C3—C8—O1	−12.3 (4)	C24—P1—C12—C17	4.6 (3)
C2—C3—C8—C7	166.3 (3)	C24—P1—C18—C19	−92.1 (2)
C3—C4—C5—C6	0.6 (4)	C24—P1—C18—C23	87.0 (2)
C4—C3—C8—O1	170.6 (3)	C24—C25—C26—C27	0.6 (4)
C4—C3—C8—C7	−10.7 (4)	C25—C24—C29—C28	0.3 (4)
C4—C5—C6—C7	−4.8 (5)	C25—C26—C27—C28	−0.2 (5)
C5—C6—C7—C8	0.9 (5)	C26—C27—C28—C29	−0.2 (5)
C6—C7—C8—O1	−174.3 (3)	C27—C28—C29—C24	0.2 (4)
C6—C7—C8—C3	6.9 (4)	C29—C24—C25—C26	−0.7 (4)

### Hydrogen-bond geometry (Å, º)

**Table:**

*D*—H···*A*	*D*—H	H···*A*	*D*···*A*	*D*—H···*A*
C9—H9*C*···O2^i^	0.98	2.69	3.439 (5)	133

Symmetry code: (i) −*x*+1, −*y*+1, −*z*.
